# Crabs ride the tide: incoming tides promote foraging of Giant Mud Crab (*Scylla serrata*)

**DOI:** 10.1186/s40462-023-00384-3

**Published:** 2023-04-17

**Authors:** Daniel E. Hewitt, Daniel D. Johnson, Iain M. Suthers, Matthew D. Taylor

**Affiliations:** 1 Fisheries and Marine Environmental Research Lab, Centre for Marine Science and Innovation, School of Biological, Earth and Environmental Science, University of New South Wales, NSW, Sydney, 2052 Australia; 2 New South Wales Department of Primary Industries, Port Stephens Fisheries Institute, NSW, Locked Bag 1, Nelson Bay, 2315 Australia; 3 Sydney Institute of Marine Science, Mosman, NSW Australia

**Keywords:** Acoustic telemetry, Accelerometry, Animal tracking, Fisheries, Habitat-use, Hidden Markov model, Animal behaviour, Crustacean, Catchability, Integrated Marine Observing System

## Abstract

**Background:**

Effective fisheries management of mobile species relies on robust knowledge of animal behaviour and habitat-use. Indices of behaviour can be useful for interpreting catch-per-unit-effort data which acts as a proxy for relative abundance. Information about habitat-use can inform stocking release strategies or the design of marine protected areas. The Giant Mud Crab (*Scylla serrata*; Family: Portunidae) is a swimming estuarine crab that supports significant fisheries harvest throughout the Indo-West Pacific, but little is known about the fine-scale movement and behaviour of this species.

**Methods:**

We tagged 18 adult Giant Mud Crab with accelerometer-equipped acoustic tags to track their fine-scale movement using a hyperbolic positioning system, alongside high temporal resolution environmental data (e.g., water temperature), in a temperate south-east Australian estuary. A hidden Markov model was used to classify movement (i.e., step length, turning angle) and acceleration data into discrete behaviours, while also considering the possibility of individual variation in behavioural dynamics. We then investigated the influence of environmental covariates on these behaviours based on previously published observations.

**Results:**

We fitted a model with two well-distinguished behavioural states describing periods of inactivity and foraging, and found no evidence of individual variation in behavioural dynamics. Inactive periods were most common (79% of time), and foraging was most likely during low, incoming tides; while inactivity was more likely as the high tide receded. Model selection removed time (hour) of day and water temperature (°C) as covariates, suggesting that they do not influence Giant Mud Crab behavioural dynamics at the temporal scale investigated.

**Conclusions:**

Our study is the first to quantitatively link fine-scale movement and behaviour of Giant Mud Crab to environmental variation. Our results suggest Giant Mud Crab are a predominantly sessile species, and support their status as an opportunistic scavenger. We demonstrate a relationship between the tidal cycle and foraging that is likely to minimize predation risk while maximizing energetic efficiency. These results may explain why tidal covariates influence catch rates in swimming crabs, and provide a foundation for standardisation and interpretation of catch-per-unit-effort data—a commonly used metric in fisheries science.

**Supplementary Information:**

The online version contains supplementary material available at 10.1186/s40462-023-00384-3.

## Background

Animal movement is a fundamental ecological process whereby individual-level behaviours (e.g., foraging, migration) give rise to emergent properties of populations (e.g., growth rates, abundance, distribution; [1]). For mobile species, quantifying individual movement underpins effective fisheries management (reviewed in [2, 3]). For example, catch-per-unit-effort (or catch rate) data is commonly used as an index of abundance in stock assessment, assuming the probability of capture (i.e., catchability) is constant for all individuals [4, 5]. However, this is rarely the case and catchability is closely linked to patterns in foraging, especially in fisheries that employ baited gear [6]. For endothermic species, foraging is typically promoted by warmer temperatures due to increased metabolism [6]. To account for this and ensure catch rate data is a reliable index of abundance, so-called ‘catch rate standardization’ (i.e., some form of regression between catch rate and environmental covariates) must be undertaken to remove the effect of environmental variation [4, 5]. As such, identifying specific behaviours (e.g., foraging; [7, 8]) and quantifying the influence of environmental variation on them can be useful in standardization and interpretation of catch-per-unit-effort data [9]. Furthermore, assessing how these behaviours are allocated among different habitats can be used to inform the design of marine protected areas [10, 11] and abundance surveys, prioritize restoration of degraded habitats [12, 13] and target release locations for stocking of hatchery reared individuals.

Acoustic telemetry is a powerful method for quantifying the movement of marine organisms and has been broadly applied in fisheries research [2, 3, 14]. Recently, hyperbolic positioning systems have allowed researchers to track animals at increasingly fine spatiotemporal scales (e.g., metres and minutes; see *Hyperbolic positioning* for technical details; [15–17]). In addition, accelerometry has enabled detailed insight into the behavioural structure of animal movement [18, 19]. These technological advances have been accompanied by a proliferation of novel statistical approaches to analyze such data [20–22]. Hidden Markov models are stochastic time-series models that classify observed animal movement or accelerometry data into unobserved (or ‘hidden’) states, which can be interpreted as proxies for behavioural states of the tagged animal [23–25]. These models are a natural choice for analyzing animal movement [23, 25] and accelerometry [24], since they explicitly model the serial dependence structure that is typical of such data. Furthermore, hidden Markov models are highly flexible, and can be extended to model the influence of environmental covariates on animal behaviour (e.g., water temperature; [26]) while accounting for individual-level variation in behaviour (e.g., [27–29]).

The Giant Mud Crab (*Scylla serrata*) is a large portunid crab (Family: Portunidae) that is widely distributed throughout the Indo-West Pacific [30]. Crabs within this family are commonly referred to as the ‘swimming crabs’, due to their efficient swimming ability afforded by flattened paddles on their fifth legs (i.e., pleopods or swimmerets; [31]). In Australia, the species supports seasonal commercial and recreational fisheries [32, 33], with the main method of harvest being the deployment of baited traps (or pots; [34, 35]) during the austral spring–summer (Hewitt et al., *unpublished data*). Adult crabs inhabit sub- and intertidal habitats in estuaries, such as mudflats and mangroves [36, 37]. As a predominantly sessile species, daily movements occur at fine spatial scales (e.g., 219–910 m d^−1^; [38]) with high site fidelity [36], however adults are capable of long distance migrations (e.g., ~ 30–200 km; [39–41]). Adult crabs are carnivorous, opportunistic scavengers [42–44] and are generally thought to forage nocturnally [38, 43] using a combination of olfaction and contact chemoreception [44, 45]. Foraging is promoted by warmer temperatures (i.e., 25–30 °C; [46]), and stable isotope analysis suggests they derive their nutrition from a combination of seagrass [47, 48], mangrove [49] and saltmarsh habitats [48, 50]. Finally, for species that inhabit shallow waters, the tidal cycle imposes frequent changes in local conditions (e.g., the availability of intertidal foraging habitat). Tidal currents may also promote movement via selective tidal-stream transport [51]; a behaviour whereby individuals use tidal currents/flow to minimize the energetic costs associated with movement [52] that has been exhibited by Giant Mud Crab [39, 41, 53].

In this study, we sought to investigate the environmental drivers of fine-scale movement and behaviour of free-ranging adult Giant Mud Crab in a creek adjoining a temperate southeast Australian estuary. Specifically, this was achieved by: (1) tracking the movement of adult crabs with accelerometer-equipped acoustic tags using a hyperbolic positioning system; and (2) using a hidden Markov model to classify movement and acceleration data into discrete behaviours, and model transitions between these behaviours as a function of environmental covariates [24, 25].

## Methods

### Study site and array design

**Fig. 1:**
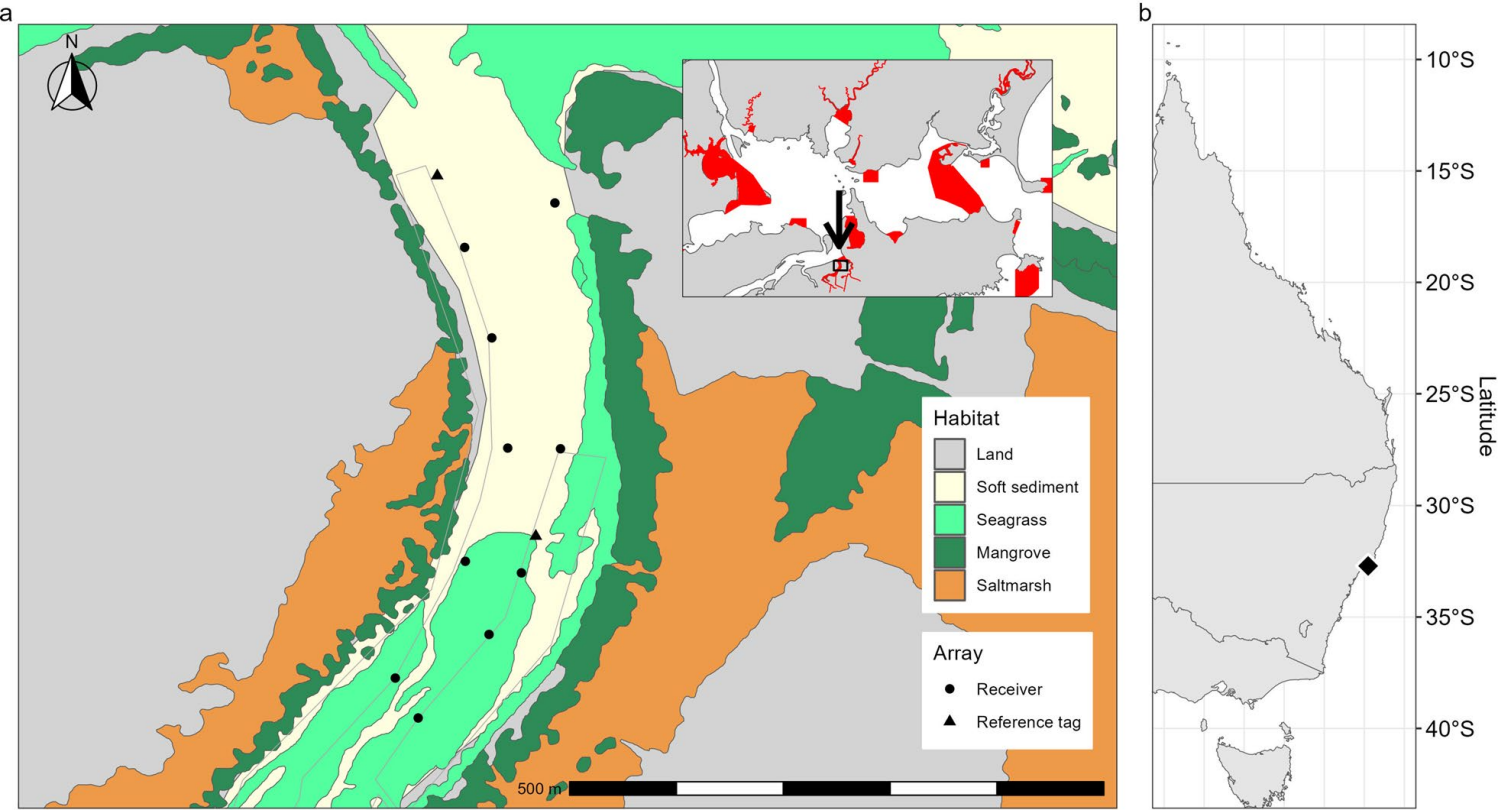
Map of **a** Fenninghams Island Creek showing the locations of receivers and fixed-position reference tags within the array, as well as the distribution of seagrass, mangrove and saltmarsh. Oyster farming infrastructure is indicated by the grey outline. The location of Fenninghams Island Creek and within Port Stephens (inset; Sanctuary Zones in red), and **b** on the east Australian coast is indicated

This study was conducted in Fenninghams Island Creek (32.75° S, 152.05° E), a small tributary to Port Stephens, a mature wave-dominated barrier estuary [54] situated on the temperate mid-north coast of New South Wales (NSW, Australia; Fig. 1). Fenninghams Island Creek is a narrow, relatively shallow creek (0.2–2 m depth) that encompasses typical estuarine habitats including unvegetated soft sediments (sub- and intertidal), seagrass (*Zostera* sp.), mangrove (*Avicennia marina*) and saltmarsh (*Sporobolus virginicus*, *Sarcocornia quinqueflora* and *Suaeda australis*; Fig. 1). It has a maximum tidal range of approximately 2 m, and mangrove and saltmarsh habitats are inundated twice daily (especially during spring tides). The study area is a ‘Sanctuary Zone’ within the Port Stephens Great Lakes Marine Park, which prohibits fishing or crab-trapping, allowing this study to proceed without the risk of fishing mortality or any effect of baited traps on movement. However, it is possible that some crabs may migrate in and out of the study area and be exposed to fishing in adjacent areas [39, 40]. Oyster farming is permitted, and tray cultivation is practiced along both shorelines of the creek (Fig. 1).

A hyperbolic positioning system (hereafter referred to as the ‘array’), employing 10 Innovasea VR2W receivers and co-located synchronisation (or ‘sync’) tags (Innovasea, Nova Scotia, Canada) was established along approximately 500 m of Fenninghams Island Creek (Fig. 1). Range testing of similar arrays in comparable systems (e.g., [55, 56]) informed a receiver spacing of 100–200 m. Each receiver and sync tag were chained to existing infrastructure (e.g., oyster trays, jetty), or independent moorings consisting of a float and anchor attached by chain, approximately 0.3–0.4 m from the bottom. To monitor positional error throughout the study, two fixed-position reference tags (V9-2x-BLU-3), with identical programming as tags deployed on crabs (see *Tag programming* below), except with a longer random transmission interval (240–360 s), were deployed within and immediately adjacent to the array (Fig. 1). Since the position of these tags were known, the distance between the estimated position (see *Hyperbolic positioning*) and their actual position provides an indication of the performance of the array throughout the study [17]. The reference tag adjacent to the array stopped transmitting after one day, and was missing at the conclusion of the study, so only detections from the reference tag within the array were used in subsequent analysis. Water temperature (°C) and conductivity (mS cm^−1^) was monitored throughout the duration of the study using a HOBO U24-002-C conductivity/salinity logger (Onset Computer Corporation, Massachusetts, USA).

Receivers were retrieved and downloaded using VUE software (v. 2.6.2; Innovasea, Amirix, Nova Scotia, Canada) after the estimated battery life of the last tag deployed had passed (~ 7 months). Detection data was subsequently uploaded to the Integrated Marine Observing System Animal Tracking Facility (IMOS ATF; https://animaltracking.aodn.org.au; [3]) which was also interrogated for any additional tag detections outside of our array.

### Crab capture and tagging

Giant Mud Crab were captured during the late austral summer (February 2020) using round collapsible mesh traps (i.e., pots; 0.9 m diameter × 0.27 m high), with 55 mm mesh and two semi-closed funnel entrances (0.25 × 0.05 m). Traps were deployed for approximately 24 h periods along 750 m of Fenninghams Island Creek (within and just upstream of the array). Captured crabs were cooled for 10–20 s in an ice/sea-water slurry (to decrease aggressive behaviour; following 39) and subsequently measured (to the nearest mm) for carapace length (CL; distance between the frontal notch and posterior carapace margin), sexed and moult-staged (following [57]). Only adult crabs (> 100 mm CL) that were likely to have recently moulted (i.e., post- or inter-moult; [57]) were tagged, to limit the probability of tag loss during ecdysis [58]. Innovasea V9A-2H accelerometer tags (hereafter ‘tags’; length: 43 mm, wet weight: 3.3 g; Innovasea, Nova Scotia, Canada) were affixed to the posterior carapace using instant adhesive (Loctite 406, Henkel Adhesives, Australia) which has shown tag retention of at least 3 months [39]. After tagging, crabs were gently submerged alongside the research vessel and once normal activity (e.g., attempted swimming) had resumed crabs were released within the bounds of the array. In general, negative impacts (e.g., stress, limb loss) are low for crabs handled and released in this manner [34]. During tagging, two previously tagged crabs were recaptured that had lost their tags, evidenced by adhesive present on the carapace. These crabs were re-tagged and data from the initial tags was excluded from our analysis (identified as continuous transmission from a single point), resulting in movement data from 18 crabs.

### Tag programming

Tags were programmed to emit a unique signal (69 kHz) with high power output (151 dB re 1 µPa at 1 m) at random intervals between 150 and 210 s (180 s nominal). High power output was chosen in an attempt to overcome potential signal attenuation owing to burial of crabs [38, 46, 59] and the presence of seagrass within the study site [60]. Random signal transmission times were employed to minimize potential signal overlap (i.e., code-collision) which can block detection. Tags were equipped with an accelerometer programmed to record tri-axial acceleration data, which represents a general index of activity, analogous to overall dynamic body acceleration (ODBA; [61, 62]). Measurements are transmitted as a root mean square (RMS) acceleration vector with a range 0–3.4 m s^−2^ [63]. Since Giant Mud Crab are expected to be predominantly slow-moving [38, 43, 46], acceleration data was recorded at 5 Hz (i.e., 5 samples s^−1^) over a 20 s window to capture ‘bursts’ of acceleration (H. Pedersen, *pers. comm.*). See Taylor, McPhan [63] for a discussion of accelerometer programming. Battery life was estimated 196 d at these settings.

### Hyperbolic positioning

Positions of tagged crabs were estimated by Innovasea using proprietary hyperbolic positioning algorithms. This approach estimates the position of tagged animals based on the time-difference-of-arrival (TDOA or multilateration) of detections at 3 or more receivers within an array. Assuming no measurement error, detection on a pair of receivers defines a hyperbola on which a tag may have been during signal transmission. Detection on a third receiver defines a second hyperbola, and the intersection of the two is the position of the tag [17]. Using this approach, the time of a detection is converted to distance based on signal propagation speed. Signal propagation speed was estimated via the Coppens equation [64] using measured water temperature (°C) and salinity and an assumed depth of 1.8 m, assuming ideal signal propagation (i.e., spreads spherically at a constant speed; [17]). Innovasea receiver clocks can drift by up to 4 s d^−1^ (dependent on water temperature) leading to differences in time among receiver clocks (i.e., clock skew). To account for this, detections from sync tags were used to calculate the skew between receiver clocks and synchronize detection times (see Smith [17] for technical details).

Overall, detections from our fixed-position reference tag indicated that location error within the array was low and positively-skewed for the duration of the study (Additional file 1: Fig. S1), with a mean error of 2.77 ± 9.24 m (SD) and median of 1.26 m (interquartile range = 0.78 m), and 93% of positions were within 5 m of their actual (known) location. In general, these results indicate that measurement error was low for the duration of the study.

### Data processing

All subsequent analysis was undertaken using R (v. 4.0.2; [65]). Visual inspection suggested no ‘tagging effects’ (e.g., elevated/decreased activity) were apparent in the tagged population (Additional file 1: Fig. S2), and we included detections from the first day of tracking in our analysis to preserve our sample size. In general, hidden Markov models are formulated in discrete-time, meaning they require temporally regular observations [66], but see Glennie et al. [67]. Many factors can contribute to temporally irregular observations in our study, including temporary emigration from the array, burial [59] and random transmission intervals in our tags. To accommodate this, we predicted temporally regular locations at 5, 10 and 15-min intervals by modelling crab movement as a continuous-time correlated random walk using the R package ‘crawl’ [68]. We refer readers to Johnson, London [69] for a full mathematical description of this model. Before predicting temporally regular locations, detections were split into ‘tracks’ where the interval between detections were greater than 4 times the interpolation interval (i.e., 20, 40 and 60-min). This was to ensure we did not introduce unreasonable uncertainty or bias our data by consecutively predicting locations within these longer temporal gaps, which would result in straight and constant movement [70]. Furthermore, tracks with less than 100 detections were excluded, as those with few observations can give rise to issues with numerical stability (i.e., non-convergence), and typically reveal less about behavioural state dynamics [71]. Locations were estimated using a state-space framework, allowing incorporation of measurement error in location estimates [69]. This was achieved by transforming the error (in metres) along the longitudinal and latitudinal axes, derived from the fixed-position reference tag, into a covariance matrix and approximating it with a bivariate Gaussian distribution during model fitting [69]. Locations were estimated via maximum likelihood, and thus require initial estimates of parameter values. To ensure adequate exploration of the likelihood surface and convergence (to a global maxima) we used 50 random perturbations of the initial parameter values and retained output from the model with the highest log-likelihood [72].

Since we predicted locations less frequently (i.e., every 5-, 10-, 15-min) than the random transmission interval of our tags (i.e., every 4–6 min) it was possible that there were some intervals where no acceleration data was recorded as no detection was recorded but a location was predicted. Relatively few missing values is typically not an issue when fitting hidden Markov models [23], and the missing observations did not contribute to the likelihood during model fitting.

### Behavioural state classification

Behaviour of tagged crabs was modelled using a hidden Markov model [73] via maximum likelihood using the R package ‘momentuHMM’ [72]. Hidden Markov models are stochastic time-series models with two components: an observable (possibly multivariate) state-dependent process, and an unobservable (‘hidden’) state-process [24, 25]. The state-dependent process consists of observed animal detections or metrics derived from them (e.g., step length, turning angle), while the state-process is a series of *N*-states, that are taken to represent the underlying behavioural modes of the animal [24, 25]. Two assumptions govern this model structure: (1) observations that comprise the state-dependent process are assumed to be conditionally independent, with the observation at time *t* conditional on the state at time *t*, and independent of all other states and observations; and (2) the state-process is a Markov chain, which means the probability of being in a given state at time *t* is completely determined by the state active at time *t* − 1. Transitions between states are governed by an *N* × *N* transition probability matrix, the entries in which denote the probability of switching states between time *t* and *t* + 1 [72], with entries on the main diagonal representing the probability of remaining in the same state (i.e., state-dwell probabilities; [24]). Hidden Markov models thereby link observed animal movement to unobserved (or ‘hidden’) underlying behavioural modes and provide a description of how they change through time [23–25].

In our case, observations that comprised the state-dependent processes included step length (m; i.e., distance moved) and mean acceleration (m s^−2^) between time *t* and *t* + 1 and turning angle (radians) between detections at *t* − 1, *t* and *t* + 1, where 0 radians corresponds to straight-line movement and ± π radians indicates course reversal. Step lengths and mean acceleration values were modelled using a zero-inflated gamma distribution, to account for instances where no movement occurred (i.e., step length = 0 m or acceleration = 0 m s^−2^; [72]). Note, the zero-inflated gamma distribution is defined only for non-negative real numbers (i.e., ≥ 0), and standard deviations greater than the mean reflect highly positively-skewed distributions and do not imply negative values. Turning angles were modelled using a wrapped Cauchy distribution, which is a probability distribution that results from ‘wrapping’ the Cauchy distribution around the unit circle, with a concentration parameter ranging between 0 and 1 that measures how concentrated turning angles are around the mean [72]. For each state, the mean (± standard deviation, SD) step length and acceleration was estimated using a log-link function, while turning angle mean was fixed at 0 radians (i.e., straight-line movement) and concentration was estimated using the logit-link function [72]. Initial parameter estimates were obtained using the same approach as with predicting temporally regular locations (see *Data processing*), whereby the fitting procedure was run 50 times with randomly selected initial values and output from the model with the highest log-likelihood was retained [72, 74]. A prior for the log-density of the working scale parameter distributions (***N***[0, 100]) was specified to avoid estimates near the boundary.

A central challenge when fitting hidden Markov models is deciding on the number of states (*N*) to estimate, which must be specified a priori, since traditional model selection techniques (e.g., minimizing information criteria) tend to select models that include more states than are biologically meaningful/interpretable [75, 76]. This is because state estimation is data-driven, meaning the estimated states may not correspond to a biologically meaningful behaviour, rather they provide proxies for them and require *post-hoc* interpretation [24, 25]. In this context, adding more states may simply be capturing random noise in the data rather than uncovering additional behavioural states. Pohle, Langrock [76] argue that the number of states should be chosen pragmatically, based on statistical and biological intuition. We expected Giant Mud Crab to exhibit 2–3 discrete behaviours, namely: inactivity/resting, foraging and possibly some inter-habitat migration [36, 38, 46, 77, 78], therefore we limited our analysis to two- and three-state models (i.e., *N* ϵ {2, 3}).

### Behavioural state dynamics

Individual-level variation in behaviour is common among free-ranging animals, due to true differences (e.g., animal ‘personality’; [79]), different environmental contexts [80] or as an artefact of variable deployment lengths between individuals [28, 81]. This can be accommodated by including discrete-valued random effects (e.g., sex, individual) in a mixed hidden Markov model [27–29, 80]. To do so, *K* mixtures (*K* ϵ {1, …, 4}) were included in a ‘null’ model (i.e., without any environmental covariates), with crab ID as a discrete-valued random effect. Under this formulation, each *K* represents a distinct transition probability matrix allowing for up to 4 behavioural ‘types’ among individuals [28, 82]. For *K* = 1, behavioural dynamics are assumed to be the same for all individuals (i.e., no random effects; [81]), while for *K* > 1 the behavioural dynamics of a given individual are governed by one of *K* transition probability matrices [27, 28]. It may be possible that > 4 behavioural ‘types’ exist and limiting *K* to a maximum of 4 was a heuristic choice, aimed at maximizing parsimony (i.e., assuming only a few behavioural types) and computational tractability. Following Isojunno et al. [80], these models were compared using Akaike information criteria (AIC) to select the optimal value for *K*, where the lowest value is indicative of the best fitting model [83]. Models were fit to data from each interpolation interval (i.e., 5, 10 and 15-min) and selected among based on model pseudo-residuals, which fulfil the role of normal-theory regression residuals for hidden Markov models [73].

The selected interpolation interval and random-effects structure was then used to model the influence of environmental covariates on behavioural transitions. Typically, entries within the transition probability matrix are assumed to be constant, however we relaxed this assumption and estimated the effect of a suite of time-varying environmental covariates on these probabilities (i.e., we assume the Markov chain is non-homogenous; [27]). This was achieved using a multinomial logit-link function which ensures all transition probabilities are between 0 and 1, and the rows of the transition probability matrix sum to 1 [74]. State transition probabilities were modelled as a function of water temperature (°C); an interaction term between tide height (m above Port Stephens Height Datum [PSHD]), and the difference in tide height over 15-min intervals (hereafter ∆-tide height); habitat type; and a cyclic effect of time (hour) of day. Cyclic effects were estimated via two periodic functions, $\mathrm{cos}(\frac{2\pi t}{24})$ and $\mathrm{sin}(\frac{2\pi t}{24})$, where *t* is the time (hour) of day (0–24) and 24 is the assumed daily periodicity of the function [29, 71, 84]. ∆-tide height includes information about both the direction of the tide (positive/negative values = flood/ebb tide) and the strength of tidal currents, where greater absolute values imply stronger tidal currents. Tide data was obtained from a nearby tide gauge (~ 4 km away; 32.72° S, 152.02° E) maintained by Manly Hydraulics Laboratory [85]. Habitat data was obtained from NSW Department of Primary Industries Fisheries Spatial Data Portal (https://www.dpi.nsw.gov.au/about-us/research-development/spatial-data-portal). This dataset includes information on the distribution of common estuarine habitats, including: seagrass, mangroves and saltmarsh [86], with a spatial resolution of approximately ± 2 m (G. West, *pers. comm.*). To account for edge effects around seagrass meadows [87, 88] a buffer of 1.26 m was applied (matching the median error in our array; see *Hyperbolic positioning*). All possible combinations of covariates were fit (including ‘null’ models with no covariates), however the tidal covariates were only included together. These models were compared using AIC, where the model with the lowest value was selected as the true model [83]. Stationary state probabilities were derived from the transition probability matrix and can be interpreted as the probability of exhibiting a given state for some fixed value of a covariate (i.e., when the system is in equilibrium). Finally, behavioural states at each location were estimated using the Viterbi algorithm, which derives the most likely sequence of states given the observations and fitted model [25, 73]. Model fit was again assessed by inspecting pseudo-residuals.

## Results

### Model selection and diagnostics

For all interpolation intervals (5-, 10- and 15-min), we found no evidence of individual-level variation in crab behaviour (i.e., *K* = 1 mixture had the lowest AIC). Furthermore, AIC increased with increasing *K* suggesting that it is unlikely that > 4 behavioural ‘types’ exist within the tagged population (Additional file 1: Table S1). Therefore, we modelled the influence of environmental covariates on crab behavioural dynamics using a ‘standard’ hidden Markov model (i.e., without random effects). Model pseudo-residuals indicated that data interpolated at 15-min intervals provided the best fit relative to the 5- and 10-min data (Additional file 1: Fig. S3). On this basis, we report only results from the model fit to data interpolated at 15-min intervals. Furthermore, we only report results from our two-state model, since the three-state model simply decomposed one state (‘inactive’ state, see *Crab behavioural states*) into two and would not have changed our biological interpretation. Finally, model selection for covariate inclusion indicated that the model including an interaction between tide height and ∆-tide height provided the best fit (Additional file 1: Table S2).

There was evidence of a diel cycle in behaviour not captured by this model, indicated by cyclic residual autocorrelation for both step length and acceleration with a ~ 12 h period (Additional file 1: Fig. S3b, f). However, the model that included a cyclic effect of time of day did not improve this. Ultimately, model fit was deemed adequate since hidden Markov models do not need to produce perfectly independent pseudo-residuals [24, 73] and small violations of this are generally of little concern when estimating behavioural state dynamics is the main goal of analysis [28] as is the case here.

The selected model was fit to 75 tracks from 13 individuals, ranging in size from 119 to 135 mm CL (Table 1). The length of tracks ranged from ~ 8 h–20 d, with an average of 1.5 ± 3.2 d. We found no evidence of tagging effects and only 9 of these 75 tracks included detections from a crab on the same day as tagging (further limiting any possible influence of tagging effects in our analysis). One female crab (ID = 7792) was detected in the coastal ocean (via the IMOS ATF) approximately 150 km north at the Port Macquarie offshore artificial reef (~ 31.42° S) 27 days after the last detection in our array.

**Table 1:** Biological and detection information for tagged Giant Mud Crab included in modelling

ID	Sex	CL (mm)	Release date	First detection	Last detection	Tracking duration (d)	Detections (*n*)	Predicted locations (*n*)	Missing acceleration (*n*)	Tracks (*n*)	Temperature range (°C)
7789	M	125	17 Feb	17 Feb	1 Jun	104.1	930	399	49	6	15.4–26.4
7791	M	119	24 Feb	24 Feb	4 Apr	8.9	867	362	27	4	23.6–27.1
7792	F	124	28 Feb	28 Feb	28 Feb	0.5	135	53	5	1	23.4–24.5
7793	M	124	19 Feb	21 Feb	30 Aug	191.1	21,275	6406	148	17	10.7–26.3
7795	F	124	24 Feb	24 Feb	13 Mar	17.3	1382	549	47	4	21.6–28.1
7796	M	125	25 Feb	25 Feb	27 Feb	1.8	355	149	13	2	24–28.1
7797	M	129	28 Feb	28 Feb	28 Feb	0.5	121	44	0	1	23.4–24.5
7798	M	119	28 Feb	28 Feb	30 Apr	62.5	532	234	23	4	19.0–24.4
7802	M	131	28 Feb	22 May	24 May	1.9	404	179	19	1	13.7–16.1
7804	M	135	20 Feb	20 Feb	8 Mar	16.1	867	320	18	3	23.4–26.7
7805	M	129	25 Feb	30 Mar	12 Apr	12.9	338	125	13	2	16.7–22.5
7807	F	127	25 Feb	27 Feb	2 Apr	34.6	1630	649	42	12	19.8–26.0
7808	M	130	17 Feb	17 Feb	11 May	83.4	5724	1844	19	18	13.7–28.1

### Crab behavioural states

**Fig. 2:**
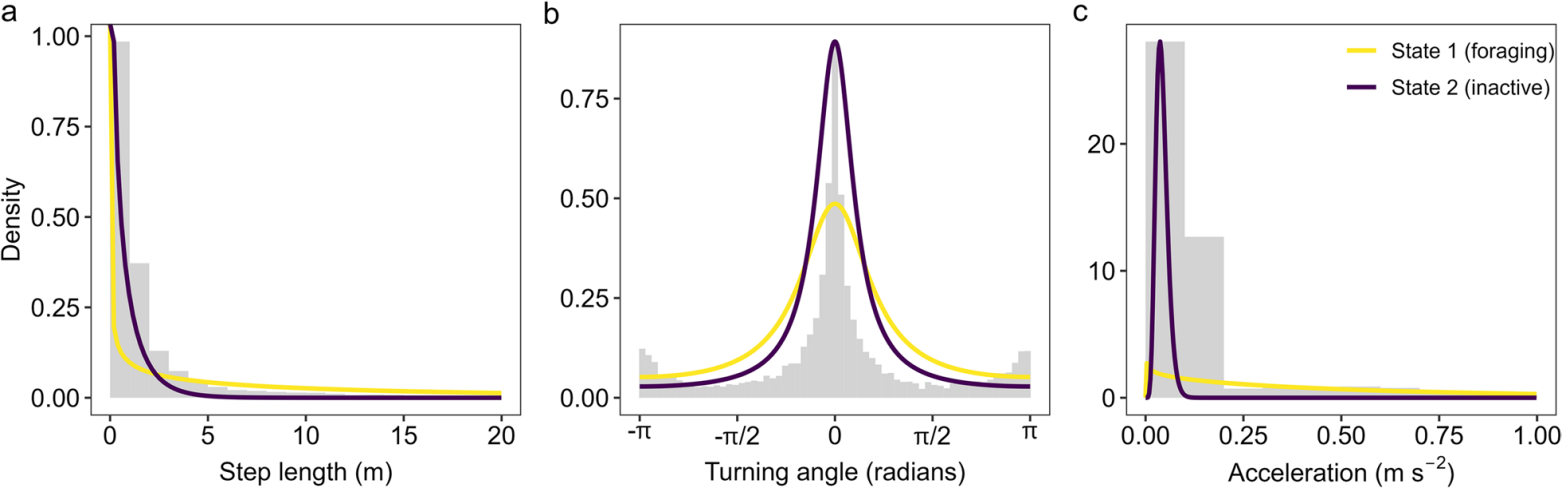
State-dependent probability distributions (lines) and histograms of observations (grey bars) for **a** step length (m), **b** turning angle (radians) and **c**) acceleration (m s^−2^). Note x-axis on (**a**, **c**) has been truncated to aid visualization and excludes the upper ~ 3% of observations

**Fig. 3:**
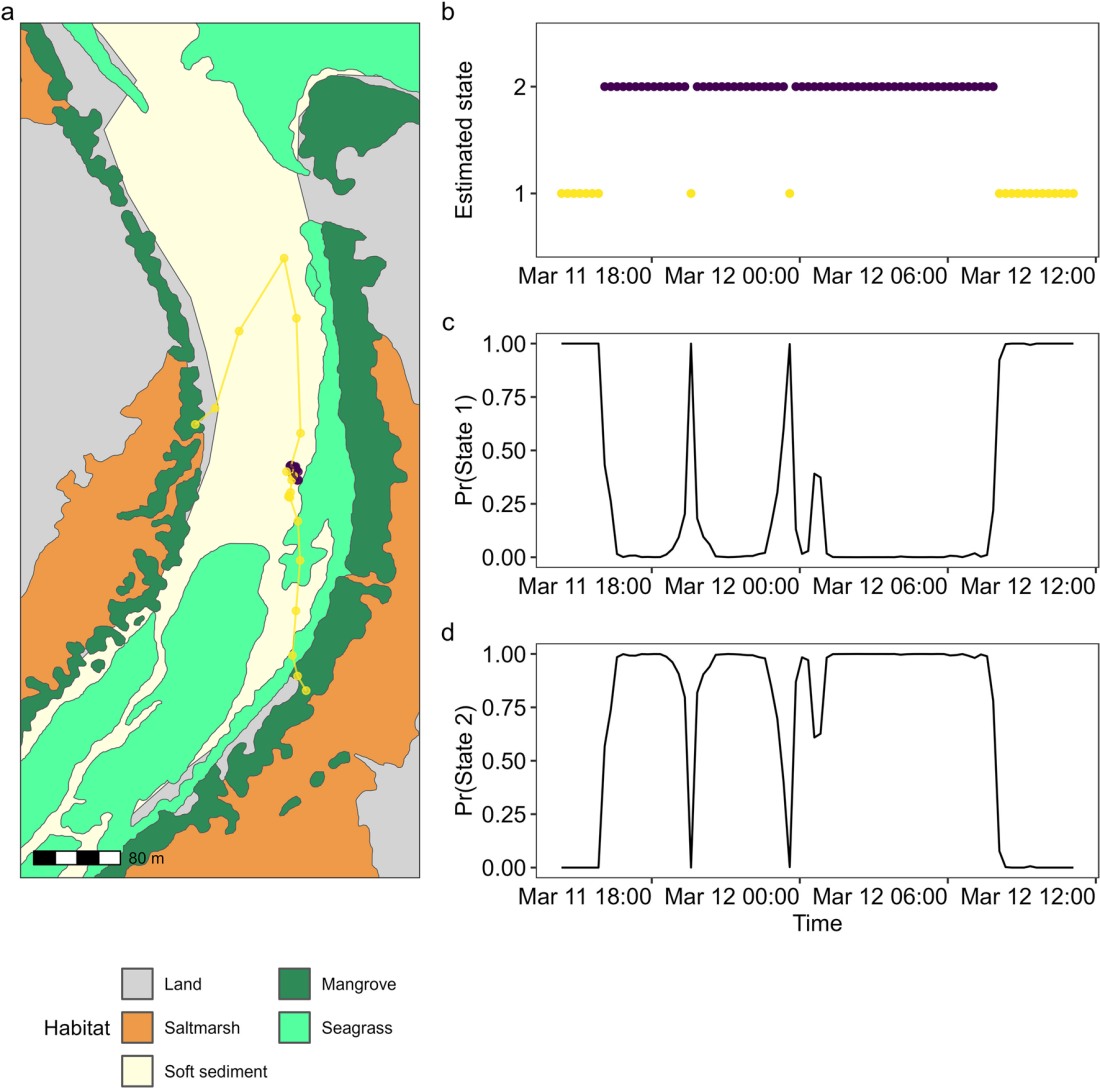
**a** Example track from a tagged female Giant Mud Crab (ID: 7795) and **b** the time-series of Viterbi-decoded behavioural states and the corresponding probabilities of **c** State 1 (foraging; yellow) and **d** State 2 (inactive; purple)

Our analysis identified behavioural states with considerable overlap in terms of their step length and turning angle concentration, however there was clear separation in terms of acceleration (Fig. 2). State 1 is likely to represent foraging (hereafter ‘foraging state’) since crabs spent little time in this state (21%) and exhibited greater, but highly variable, step.lengths (mean ± SD = 13.98 ± 18.10 m 15 min^−1^) and acceleration (0.59 ± 0.63 m s^−2^ 15 min^−1^), coupled with moderately concentrated turning angles (concentration = 0.51; Fig. 2; Table 2), indicative of a combination of straight-line movement and direction changes. In the foraging state, crabs also had the lowest probability of no movement (Table 2). However, they exhibited a higher probability of exhibiting no acceleration which is likely due to the relatively high variability in acceleration while in this state (Table 2). Crabs spent most of their time in State 2 (79%), which is likely to correspond to periods of inactivity (hereafter ‘inactive state’). Crabs in this state exhibited much shorter step lengths (mean ± SD = 0.75 ± 0.93 m 15 min^−1^), low acceleration (0.04 ± 0.01 m s^−2^ 15 min^−1^), and relatively highly concentrated turning angles (concentration = 0.70), indicative of infrequent changes in direction (Fig. 2; Table 2). An example track, with the most likely sequence of states is depicted in Fig. 3.

**Table 2:** State-dependent parameter estimates for tagged Giant Mud Crab

State	Step length (m)^a^		Turning angle (radians)	Acceleration (m s^−2^)^a^	
	Mean ± SD	Pr(0)	Concentration	Mean ± SD	Pr(0)
1	13.98 ± 18.10	<< 0.001	0.51	0.59 ± 0.63	0.006
2	0.75 ± 0.93	0.014	0.70	0.04 ± 0.02	0.002

^a^The zero-inflated gamma distribution used to model step length and acceleration is defined only for non-negative real numbers (i.e., ≥ 0), and standard deviations greater than the mean reflect highly positively-skewed distributions

### Behavioural state dynamics

**Fig. 4:**
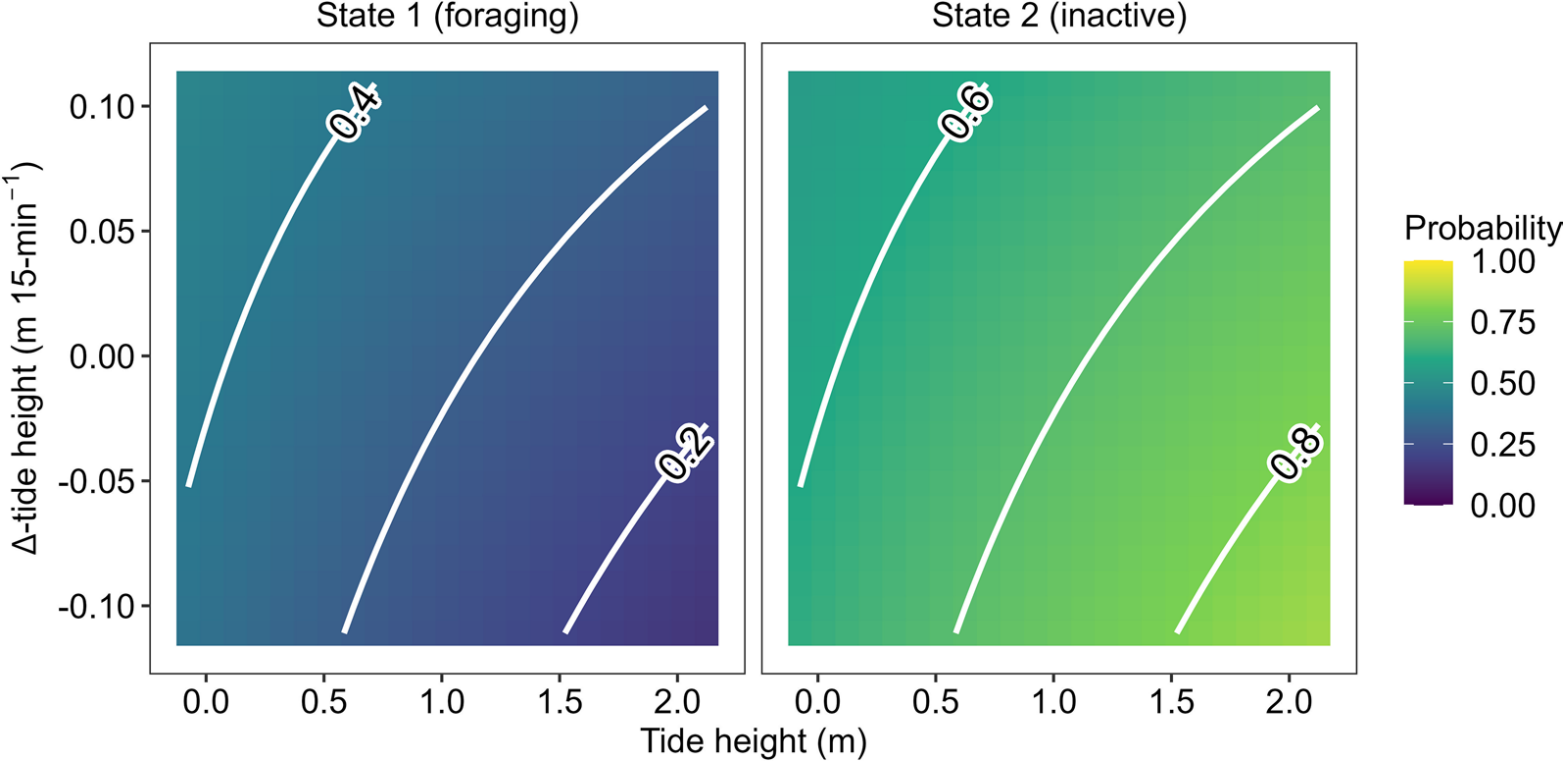
Stationary state probabilities as function of the interaction between tide height (m) and ∆-tide height (m 15-min^−1^) in tagged Giant Mud Crab

**Fig. 5:**
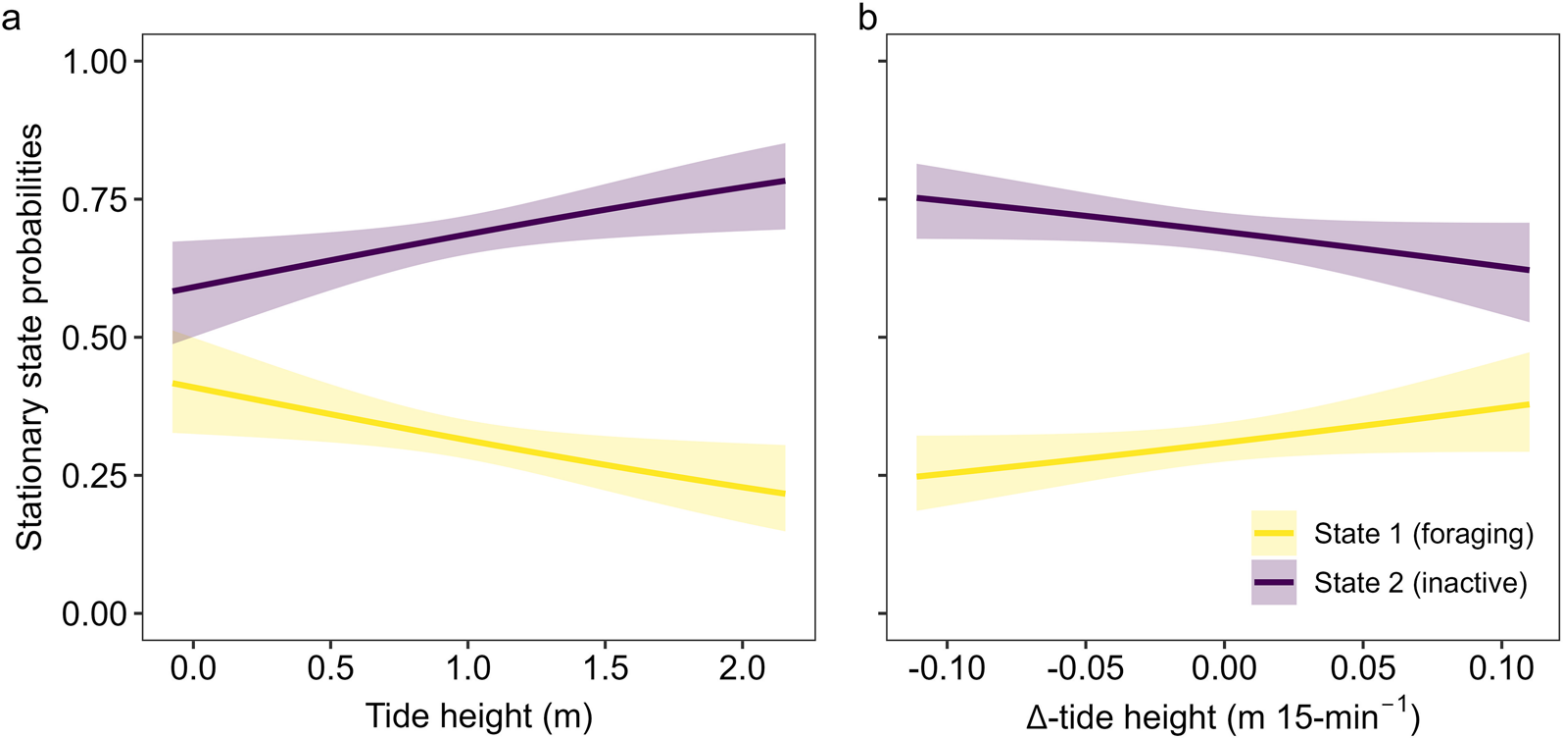
Stationary state probabilities (± 95% CI) as a function of **a** tide height (m) and **b** ∆-tide height (m 15-min^−1^)

States were highly persistent through time, indicated by very high state-dwell probabilities (i.e., diagonal entries in Table 3). Based on our model selection, water temperature (°C), time (hour) of day and habitat type were removed from our model, implying these covariates explain little about crab behavioural dynamics. The interaction between tide height (m) and ∆-tide height (m 15-min^−1^) suggested that crabs were most likely to be foraging during the low (< 0.5 m) incoming tide (Fig. 4), and more likely to be inactive as the high tide recedes (Fig. 4). Overall, crabs were always more likely to be inactive than foraging (Fig. 5), but they were approximately twice as likely to be foraging at low tide than at high (Fig. 5a). Conversely, crabs were approximately 1.25 times more likely to be inactive at high tide relative to low (Fig. 5a). The probability of foraging was higher during an incoming tide than an outgoing tide (Fig. 5b), while the opposite is true for the inactive state which becomes more likely as the tide recedes (Fig. 5b).

**Table 3:** State transition probabilities of tagged Giant Mud Crab

Current state	Next state	
	1	2
1	0.91	0.09
2	0.04	0.96

Diagonal entries indicate the probability of staying in the same state (i.e., state-dwell probabilities)

## Discussion

Our study is the first to quantitatively link fine-scale movement and behaviour of Giant Mud Crab to environmental variation. This was achieved using high spatial and temporal resolution, accelerometer-equipped acoustic tags and high temporal resolution environmental data. Modelling data with a hidden Markov model allowed the classification of observations and provided insight into the fine-scale drivers of behaviour for the species. Our analysis shows that the tidal cycle is an important driver of foraging, that allows crabs to minimize visual predation risk, and optimize the energetic efficiency of foraging. Quantifying movement and behaviour of mobile exploited species is important for developing effective fisheries management [2, 3], and our results contribute to the evidence base that underpins management actions for this species*.*

### Fine-scale movement and behaviour of Giant Mud Crab

Direct observation of aquatic animal behaviour is challenging, especially in turbid estuarine waters. The main advantage of hidden Markov models when applied to animal movement is the classification of observed movement and acceleration into ‘states’ that may correspond to biologically meaningful behaviours [23–25]. In our analysis, Giant Mud Crab were inactive for a majority of the time (79%), which shows they are a predominantly sessile species [38, 43, 46]. Adult Giant Mud Crab are considered opportunistic scavengers [42], and foraging is facilitated via a combination of olfaction [45] and contact chemoreception [44]. Crabs generally exhibit an initial ‘searching’ response towards olfactory cues [45] to find the approximate location of the food [78], followed by further tactile investigation using the dactyls of the walking legs to find the exact location of the prey/food item [44]. This description of foraging is well explained by the foraging state in our model, which includes a combination of long and short movements with higher overall activity (i.e., acceleration), and variability in terms of directional persistence.

For aquatic species that inhabit shallow-water habitats, the semidiurnal (i.e., twice daily) tidal cycle imposes a regular change in the prevailing conditions [51]. We found that Giant Mud Crab are most likely to forage when the tide is low (< 0.5 m) and incoming, while they are most likely to be inactive on an outgoing, high tide. This likely reflects exploration of shallow or intertidal foraging habitat (e.g., mangroves, mudflats; [49, 77]) as the tide is rising and they become inundated. Larger predators (e.g., *Carcharhinus leucas*, Bull Shark) are unlikely to be able to access these habitats when water levels are shallower, thereby lowering predation risk—a strategy employed by other estuarine species (e.g., *Acanthopagrus australis*, Yellowfin Bream; 63). Similarly, the high probability of inactivity during high tide likely reflects a predator avoidance strategy, since Giant Mud Crab typically bury in the mud during periods of inactivity [38, 46]. This description of foraging behaviour closely matches observations of the distribution of Giant Mud Crab in an intertidal region in a nearby estuary (Moreton Bay, Queensland; [77]). Additionally, foraging during strong incoming tides may also be indicative of the use of selective tidal-stream transport [52], a behaviour exhibited by the species elsewhere [39, 41, 53]. If crabs use incoming tides to facilitate movement this may decrease the energetic cost of foraging [52], thereby minimizing the energetic trade-off of searching for prey [89]. However, this may come at the cost of being able to efficiently sample olfactory queues, since swimming with the current is likely to result in a crab remaining in the same olfactory ‘patch’.

In general, warmer temperatures promote foraging in Giant Mud Crab [46]. This is likely due to increased metabolism [90], which may increase their motivation to feed and energetic requirements [6, 91]. However, our analysis suggests that water temperature is not an important driver of fine-scale movement and behaviour of Giant Mud Crab. It is likely that the results presented in Hill [46] represent the seasonal influence of temperature (since they included a period of acclimation) on Giant Mud Crab behaviour, while our analysis is aimed at a much finer temporal resolution (e.g., observations every 15-min over several days) over which the variation of estuarine water temperature is comparatively low. Future studies could employ longer random transmission intervals in tags, thereby preserving battery life and enabling inter-seasonal tracking of individuals. Such data may be amenable to analysis using a ‘hierarchical’ hidden Markov model, which models state processes that operate at different timescales [92, 93], provided the assumed dependence structure is appropriate (see Glennie et al. [67]).

We did not find any evidence of nocturnal foraging, which is thought to represent a visual-predator avoidance strategy and has previously been reported for the species [38, 43]. However, in highly turbid waters visual predation is somewhat reduced and overall predation pressure is likely to be much more diffuse. This may explain the lack of diel rhythm in Giant Mud Crab behaviour in the present study, and elsewhere (e.g., [94]). Similarly, our analysis suggests that habitat type has little influence on crab behavioural dynamics, reflecting their status as opportunistic scavengers [42–44]. This is further supported by several stable isotope studies that saltmarsh grass (i.e., *Sporobolus virginicus*; [48, 50]), seagrass [47] and mangroves [49] all contribute to Giant Mud Crab nutrition. Ultimately, stable isotopes provide an indication as to which habitats form the base of an animals diet, and it is likely that crabs tagged in the present study are carnivorous; feeding on benthic macroinvertebrates (e.g., gastropods, crustaceans and molluscs; 43) that are primary consumers across these habitats.

### Implications for fisheries management

Quantifying drivers of animal movement is important for effective fisheries assessment, and management that relies on it [2, 3]. For example, catch-per-unit-effort data is assumed to represent an index of relative abundance, and forms the basis of most contemporary stock assessments [95]. However, use of catch-per-unit-effort as an index of abundance assumes that catchability of target individuals is constant [4, 5], and it is important to consider how environmental variation (e.g., low temperatures) influences foraging and responding to baited traps [6], when standardizing and interpreting catch rates [5]. Our results suggest that the tidal cycle is closely related to patterns in foraging, which could influence catchability, making it an important covariate to consider for catch rate standardisation, as is the case for the closely related Blue Swimmer Crab (*Portunus armatus*; [96]). However, crab fishers may deploy traps for several days at a time, which may lead to an apparent decoupling of the relationship between catchability in the tidal cycle in catch data. While our analysis suggests water temperature does not affect fine-scale behaviours, many studies have shown a strong temperature effect on Giant Mud Crab catch rates [32, 97] and it is likely that this is still an important covariate to include in catch rate standardization.

Several fisheries management strategies require information about the partitioning of time and behaviours among habitats. For example, stocking of hatchery-reared individuals requires that release locations support the suite of habitats required to support routine behaviours (e.g., foraging). Our analysis suggests that Giant Mud Crab may be highly adaptable in this regard, since they did not exhibit a clear preference for foraging in a particular habitat. However, overall productivity of the system must also be considered (e.g., [90, 98]), and specific habitats (e.g., seagrass) may confer other benefits (e.g., enhanced survival of juveniles) that are not considered in our analysis [99].

### Technical considerations and caveats

Tag loss is an important concern in acoustic tagging studies [100, 101], especially when externally tagging crustaceans, as the exoskeleton will be shed during ecdysis [58]. In our study, this was avoided by only tagging large, recently moulted individuals (see *Crab capture and tagging*) and our approach was largely successful, resulting in only two tag loss incidents. These were likely a consequence of re-entering a trap rather than ecdysis, as glue was present on the carapace of these individuals and they were still in ‘hard-shell’ condition [57] when recaptured. Ultimately, the number of tagged crabs was within the range appropriate for making behavioural inferences at a population scale [101].

Recently, ‘tagging effects’ (e.g., elevated activity) have been documented in other crab species (e.g., Snow Crab, *Chionoecetes opilio*), which is typically dealt with by discarding detections from the first day of tracking [102, 103]. While we found no evidence of changes in activity, tagging effects in crabs may manifest themselves in ways not amenable to visual inspection of activity, such as burial (i.e., seeking refuge) or emigration from the area. However, our analysis requires relatively long series of consecutive detections (≥ 100), permitting only small interruptions (≤ 1 h) otherwise the data is excluded, and it is likely burial, or emigration would violate these conditions. Furthermore, catch-and-release does not typically induce high levels of stress in the species [34] and previous tagging studies have not found evidence of tagging effects [38, 39] giving us confidence that they have not influenced the analysis presented here.

The importance of accounting for individual-level variation in behaviour is increasingly being recognized in animal tracking studies [79]. This variation can be due to true differences (i.e., animal ‘personality’; [79]), variable deployment lengths and different (unmeasured) environmental contexts encountered by tagged individuals [28]. In our analysis, we found no evidence of individual-level variation in fine-scale behavioural dynamics of Giant Mud Crab. This may be because we only tagged large, adult crabs (119–135 mm CL) that had recently moulted or because our sample size was low relative to the frequency that individual variation is exhibited within the population. It is possible to modify this approach to account for sex-specific differences in behaviour (e.g., [29]), however this approach typically requires larger sample sizes to be reliable [81] and previous studies have not detected any differences in fine-scale behaviour between sexes [46]. Conversely, at greater temporal scales (e.g., seasonal) differences in movement have been observed. For example, mature female Giant Mud Crab typically migrate to oceanic waters to spawn [39, 40, 53], facilitating the broad-scale dispersal of larvae [104] which may explain the detection of a tagged female ~ 150 km north of our array in the coastal ocean. While males are typically thought to remain within estuaries, there have been a few examples of broad-scale migrations reported [41].

Measurement error within our array was generally low (median = 1.26 m) and consistent with inherent GPS error (2–3 m; [17]), which was used to define the ‘known’ positions of receivers and reference tags. Additionally, some error may have been due to the presence of structurally complex habitats such as seagrass, mangroves and oyster farming infrastructure, which can lead to issues with signal attenuation [60], refraction, or reflection (sometimes refered to as ‘multipath’; [105]). Measurement error can increase the overlap between state-dependent distributions (e.g., similar step lengths) which may lead to difficulty differentiating states [106]. The inclusion of acceleration—which is not subject to any location measurement error—buffers against this in our analysis, and acceleration within the foraging state was markedly different from the inactive state. Despite this, some state misclassification may have still occurred. For example, we would expect crabs in the inactive state to have relatively low concentration in their turning angles, given consecutive location estimates around the true location. However, the relatively high concentration in this state may imply that some very fine-scale (i.e., < 1 m), low-acceleration foraging movements (e.g., scavenging on a fish carcass) were assigned to the inactive state, or that resting crabs may drift in currents to some extent. State classification can be further aided by fitting hidden Markov models in a semi-supervised context via the incorporation of ‘known’ (or labelled) states, which are typically derived from laboratory observations [24]. However, movements in a laboratory setting may not be representative of free-ranging animals [24, 107] and incorporation of labelled data can be practically and computationally challenging (V. Leos-Barajas, *pers. comm.*).

Finally, acoustic tags must be submerged to record detections, and while it is possible for Giant Mud Crab to spend prolonged periods out of the water, this is uncommon for adults in the size range tagged [77] giving us confidence that this did not exert undue influence on the results presented here.

## Conclusions

Our description of Giant Mud Crab behavioural dynamics are in close agreement with observations of Giant Mud Crab behaviour [43–46] and qualitatively similar to a previous active-tracking study (that did not record/report any environmental data; [38]). Furthermore, these results provide a mechanistic explanation of the observed distribution of the species across sub–intertidal habitats [77]. We demonstrate the importance of the tidal cycle in driving foraging of Giant Mud Crab, likely as a strategy to minimize predation and maximize energetic efficiency, similar to other estuarine species [63]. Determining such relationships adds to the evidence base supporting fisheries management [2, 3] and the patterns resolve aid the standardisation and interpretation of catch-per-unit-effort data.

## Supplementary Information

**Additional file 1. Supplementary Figure 1.** Daily horizontal position error (m) of a fixed-position reference tag within our array. Horizontal position error is the difference between the known position of the tag and its estimated position. The y axis in a) is truncated to show the majority of the data (i.e., 95 % of detections < 10 m horizontal position error), while b) shows all detections.

## Data Availability

The datasets generated and/or analyzed during the current study can be accessed through the IMOS ATF (https://animaltracking.aodn.org.au) under the NSW DPI Coastal and Estuarine Fish Tracking (CEFT) project. The code used for analysis can be accessed via GitHub (https://github.com/DEHewitt/gmc_vps_hmm) or upon reasonable request to the corresponding author. The hyperbolic positioning algorithms used are the property of Innovasea and are unavailable at the time of publication.

## Abbreviations

CL: Carapace length
ODBA: Overall dynamic body acceleration
RMS: Root mean square
SD: Standard deviation
TDOA: Time-difference-of-arrival
∆-tide height: Difference in tide height in 15-min intervals
