# Supplementary material for: Crabs ride the tide: incoming tides promote foraging of Giant Mud Crab (Scylla serrata)
Source: Mov Ecol. 2023 Apr 17;11:21. doi: 10.1186/s40462-023-00384-3 (PMC10108527; doi:10.1186/s40462-023-00384-3)
Supplement: Supplementary file 1 — Additional file 1. Supplementary Figure 1. Daily horizontal position error (m) of a fixed-position reference tag within our array. Horizontal position error is the difference between the known position of the tag and its estimated position. The y axis in a) is truncated to show the majority of the data (i.e., 95 % of detections < 10 m horizontal position error), while b) shows all detections. [file 40462_2023_384_MOESM1_ESM.docx]

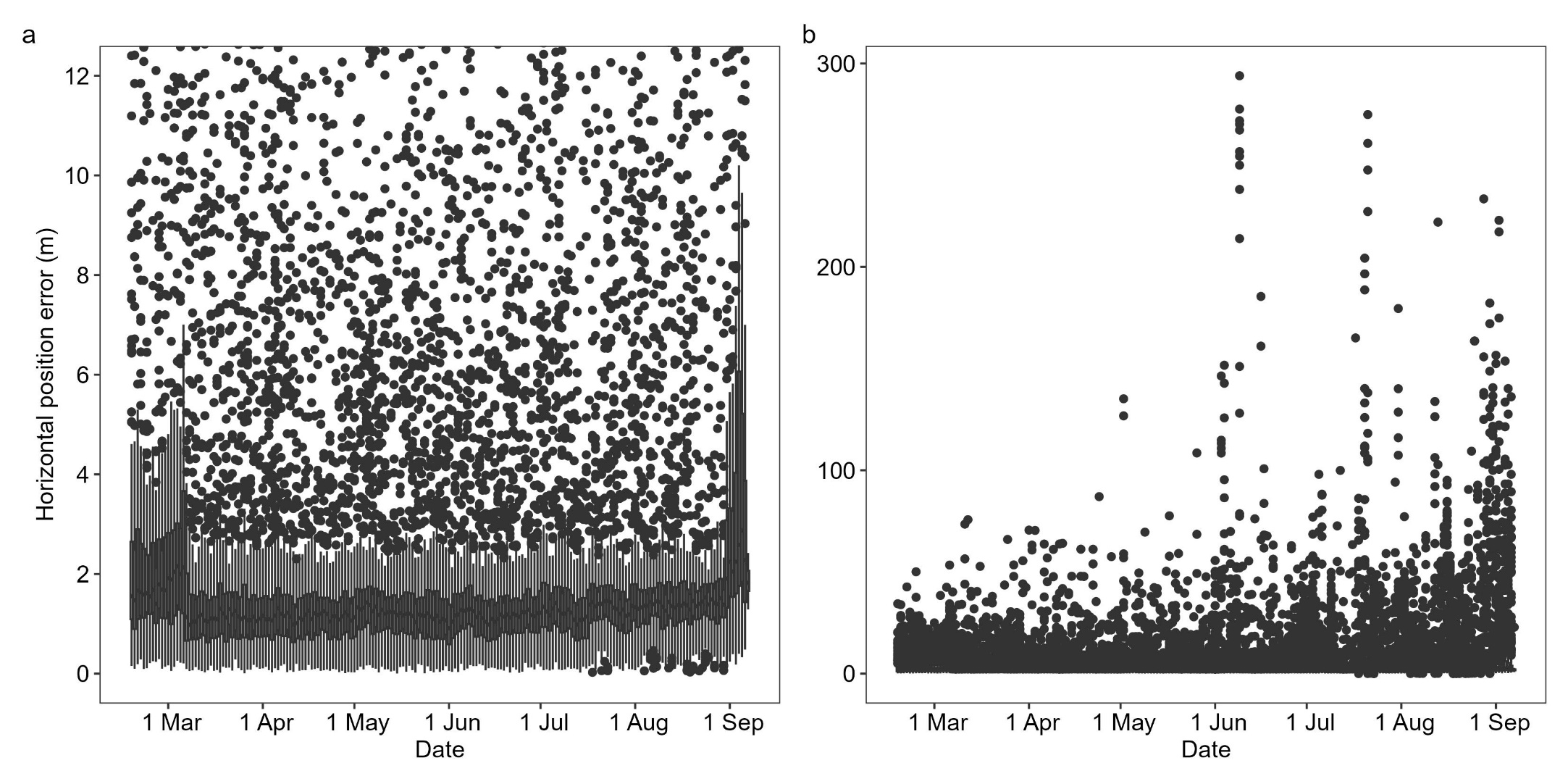


**Supplementary Figure 1.** Daily horizontal position error (m) of a fixed-position reference tag within our array. Horizontal position error is the difference between the known position of the tag and its estimated position. The *y*-axis in **a**) is truncated to show the majority of the data (i.e., 95 % of detections < 10 m horizontal position error), while **b**) shows all detections.


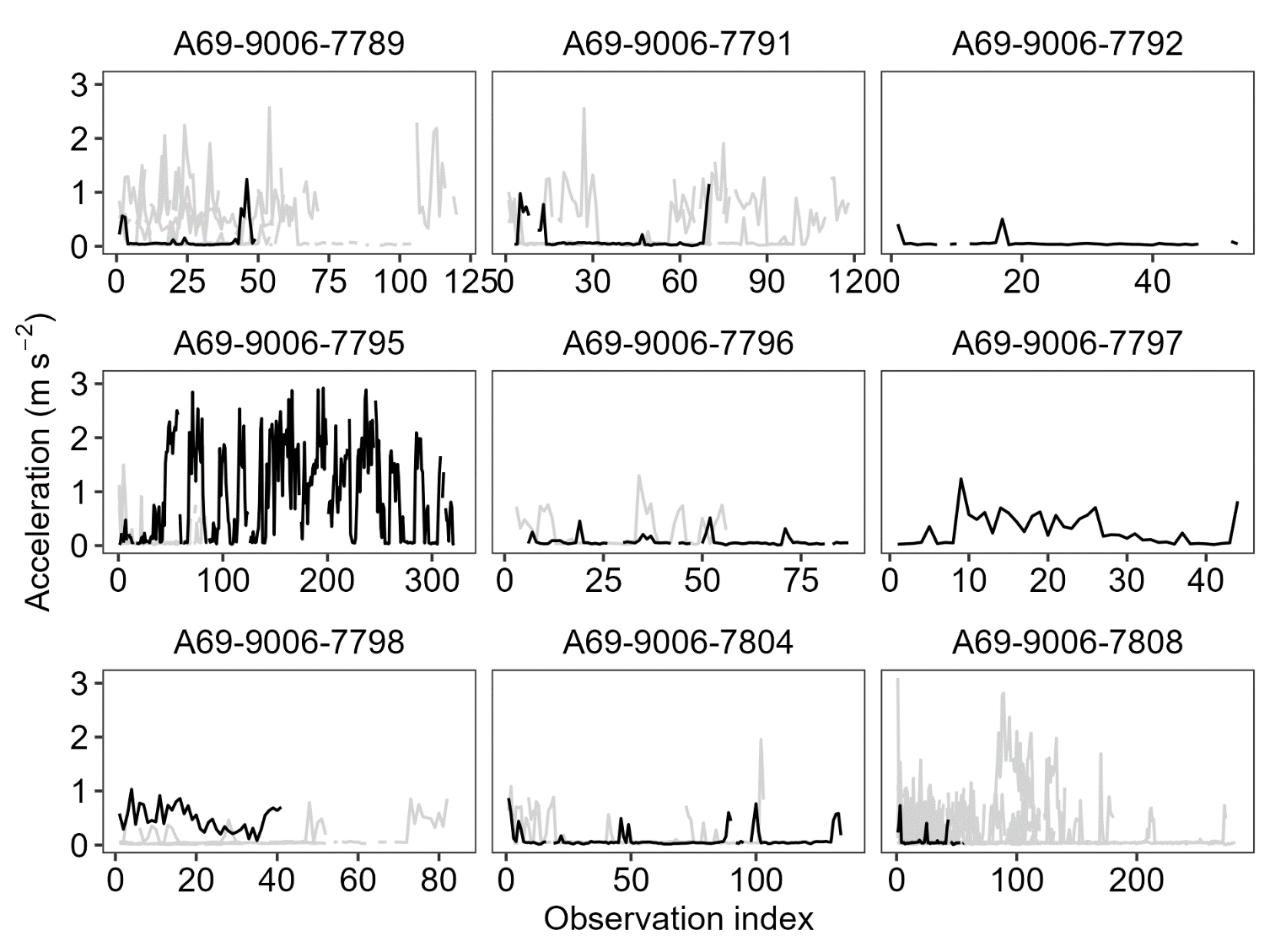


**Supplementary Figure 2.** Acceleration (m s^-2^) of tagged crabs that were detected on the same day as tagging. The black line corresponds to detections on the first day of tracking while the grey lines representall other ‘tracks’. For an explanation of ‘tracks’ see *Data processing* in main text.


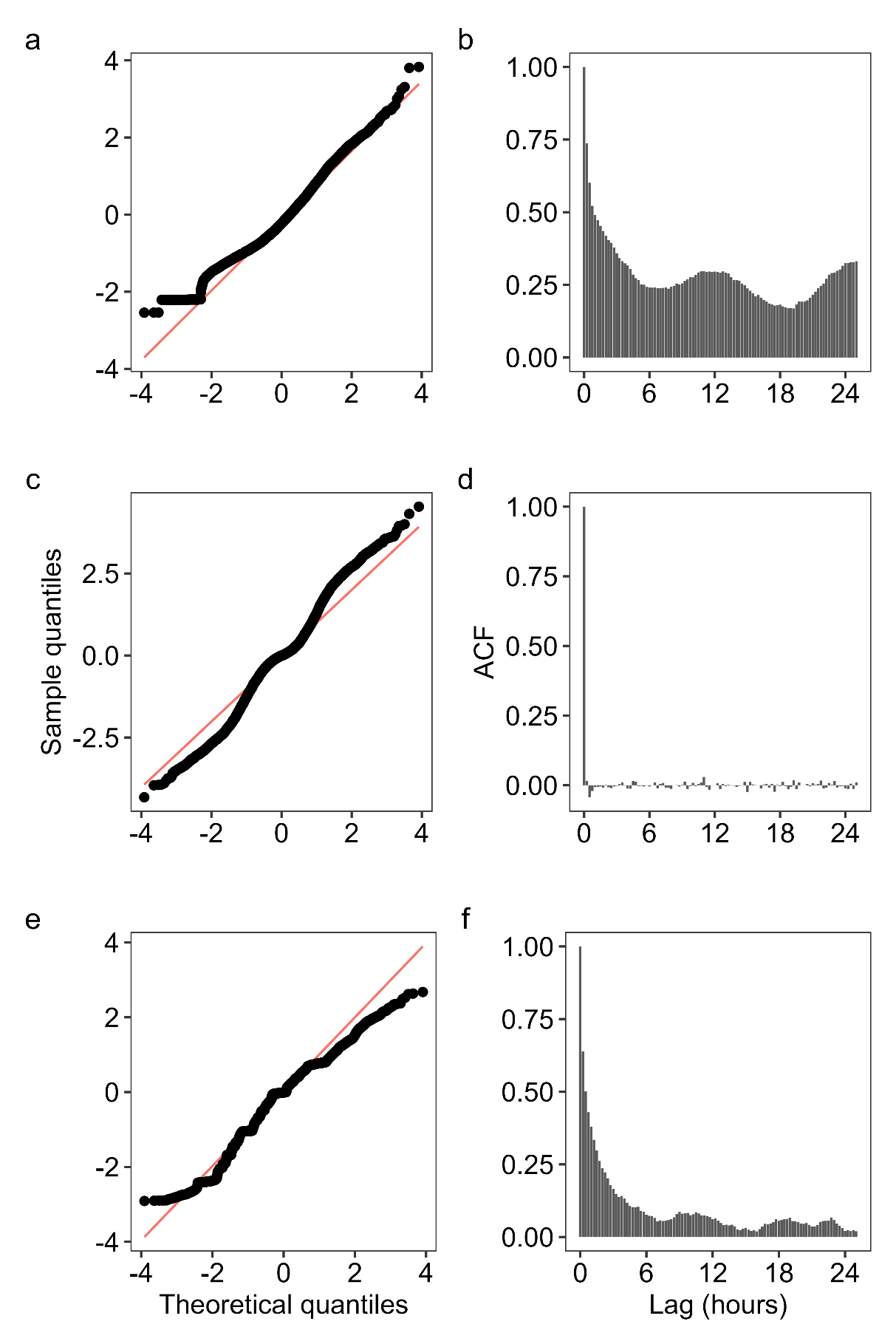


**Supplementary Figure 3.** Model pseudo-residuals and associated autocorrelation functions from our 2-state hidden Markov model for step length (**a**, **b**), turning angle (**c**, **d**) and acceleration (**e**, **f**).

**Supplementary Table 1.** Model selection results comparing ‘standard’ (i.e., *K* = 1) and mixed effects (i.e., *K* > 1) hidden Markov models fit to data interpolated at 5-, 10- and 15-min intervals.

| Number of mixtures (*K*) | Parameters (*n*) | ∆AIC |
| --- | --- | --- |
| 5-min interpolation | | |
| 1 | 17 | 0.00 |
| 2 | 47 | 3,503.92 |
| 3 | 32 | 3,752.87 |
| 4 | 62 | 11,544.65 |
| 10-min interpolation | | |
| 1 | 17 | 0.00 |
| 2 | 33 | 2,155.06 |
| 3 | 49 | 4,325.44 |
| 4 | 65 | 6,213.82 |
| 15-min interpolation | | |
| 1 | 17 | 0.00 |
| 2 | 33 | 1,035.115 |
| 3 | 49 | 1,642.35 |
| 4 | 65 | 7,239,63 |

**Supplementary Table 2.** Model selection for covariate inclusion in a hidden Markov model fit to data interpolated at 15-min intervals.

| Covariates | Parameters (*n*) | ∆AIC |
| --- | --- | --- |
| Tide height × ∆-tide height | 23 | 0.00 |
| TOD | 21 | 49.16 |
| Habitat | 23 | 226.89 |
| No covariates | 17 | 278.22 |
| Water temperature | 19 | 486.16 |
| TOD + water temperature | 23 | 653.62 |
| TOD + habitat | 27 | 732.53 |
| Habitat + water temperature | 25 | 1,055.30 |
| TOD + tide height × ∆-tide height | 27 | 1,115.53 |
| Tide height × ∆-tide height + water temperature | 25 | 1,161.44 |
| Habitat + tide height × ∆-tide height | 29 | 1,393.35 |
| TOD + habitat + water temperature | 29 | 1,613.24 |
| TOD + tide height × ∆-tide height + water temperature | 29 | 1,633.87 |
| All covariates | 35 | 1,813.34 |
| Habitat + tide height × ∆-tide height + water temperature | 31 | 1,976.57 |
| TOD + habitat + tide height × ∆-tide height | 33 | 2,262.39 |
